# Taxonomic scheme of the order Chaetophorales (Chlorophyceae, Chlorophyta) based on chloroplast genomes

**DOI:** 10.1186/s12864-020-06845-y

**Published:** 2020-06-26

**Authors:** Benwen Liu, Yuxin Hu, Zhengyu Hu, Guoxiang Liu, Huan Zhu

**Affiliations:** 1 Key Laboratory of Algal Biology, Institute of Hydrobiology, Chinese Academy of Sciences, Wuhan, 430072 China; 2 University of Chinese Academy of Sciences, Beijing, 100039 China; 3 State Key Laboratory of Freshwater Ecology and Biotechnology, Institute of Hydrobiology, Chinese Academy of Sciences, Wuhan, 430072 China

**Keywords:** Chlorophyta, Classification, Gene order rearrangement, Green algae, Nuclear genes

## Abstract

**Background:**

Order Chaetophorales currently includes six families, namely Schizomeridaceae, Aphanochaetaceae, Barrancaceae, Uronemataceae, Fritschiellaceae, and Chaetophoraceae. The phylogenetic relationships of Chaetophorales have been inferred primarily based on short and less informative rDNA sequences. This study aimed to phylogenetically reconstruct order Chaetophorales and determine the taxonomic scheme, and to further understand the evolution of order Chaetophorales.

**Results:**

In the present study, seven complete and five fragmentary chloroplast genomes were harvested. Phylogenomic and comparative genomic analysis were performed to determine the taxonomic scheme within Chaetophorales. Consequently, Oedogoniales was found to be a sister to a clade linking Chaetophorales and Chaetopeltidales. Schizomeriaceae, and Aphanochaetaceae clustered into a well-resolved basal clade in Chaetophorales, inconsistent with the results of phylogenetic analysis based on *rDNA* sequences. Comparative genomic analyses revealed that the chloroplast genomes of Schizomeriaceae and Aphanochaetaceae were highly conserved and homologous, highlighting the closest relationship in this order. Germination types of zoospores precisely correlated with the phylogenetic relationships.

**Conclusions:**

chloroplast genome structure analyses, synteny analyses, and zoospore germination analyses were concurrent with phylogenetic analyses based on the chloroplast genome, and all of them robustly determined the unique taxonomic scheme of Chaetophorales and the relationships of Oedogoniales, Chaetophorales, and Chaetopeltidales.

## Background

Class Chlorophyceae comprises two primary lineages based on molecular phylogeny, one comprising orders Sphaeropleales and Volvocales (SV clade) and another comprising orders Oedogoniales, Chaetophorales, and Chaetopeltidales (OCC clade) [1–6]. Order Chaetophorales, a lesser known member of Chlorophyceae (Chlorophyta) first circumscribed by Wille [7], containing nine families, as reported by Printz [8] and six families, as reported by Bourrelly [9]. Based on ultrastructural studies (mitosis-cytokinesis, motile cell) and molecular phylogenetic analyses, six families (Schizomeridaceae, Aphanochaetaceae, Barrancaceae Uronemataceae, Chaetophoraceae, and Fritschiellaceae) have been reported in this order and numerous traditional families were transferred to other green algal orders or classes [10–23]. Although Chaetophorales has exhibited uncontested monophyly upon improvement in gene and taxon sampling [20, 21], certain key relationships within this order and the OCC clade are less prominent and warrant further investigation. Previous molecular phylogenetic analyses focusing on taxonomic schemes in this order simply included few species or single molecular marker, which failed to reveal relationships within Chaetophorales [1, 2, 5, 24]. The preliminary taxonomic scheme was not presented until Caisová et al. [20] reported certain additional representative species and *18S rDNA* sequences in Chaetophorales. Thereafter, family Barrancaceae, as a new member was included in Chaetophorales [21] and the broadly defined family Chaetophoraceae was revised with an additional family, i.e., Fritschiellaceae [23]. The three most common and well-known genera of Chaetophorales, i.e., *Chaetophora*, *Stigeoclonium*, and *Aphanochaete* are polyphyletic [20, 21]. Relationships among families remain unclear, indicating the need to better understand the taxonomic scheme of this order. Most of the aforementioned phylogenetic studies are based on one or a few *rRNA* genes and were performed with partial Chaetophoralean taxa, and few studies have focused on chloroplast genes and the chloroplast genome.

Thus far, only two organelle genomes have been published in Chaetophorales [25, 26], thus restricting our understanding of the taxonomic status and evolution of this group. Taxon sampling, especially the lack of important species, e.g., type species in each genus, is still the most prominent obstacle for phylogenetic analysis of Chaetophorales. Chloroplast phylogenomics has recently been considered a useful approach to elucidate enigmatic evolutionary relationships among different plant taxa [27–32]. The plastome has been increasingly applied for phylogenetic and evolutionary studies on green algae with its unique advantages. The acquisition of high-density plastid genomic data, coupled with the establishment of various complex analytical methods, could greatly help understand the evolution of green plants [31, 33–38]. This study attempted to obtain 12 chloroplast genomes in Chaetophorales. This study aimed to phylogenetically reconstruct order Chaetophorales and determine the taxonomic scheme and to further the current understanding of the evolution of the order Chaetophorales.

## Results

### General features of cpDNA

This study contains data from 14 chloroplast genomes representing the existing major branches of Chaetophorales. Seven of twelve newly added chloroplast genomes were with complete genomic maps (Additional file 1).

All complete chloroplast genomes of Chaetophorales (Table 1) consistently contained 67 protein-coding genes and 3 *rRNA* genes without inverted repeats (IR). Protein-coding genes primarily included 5 *psa*, 15 *psb*, 11 *rps*, 8 *rpl*, 6 *atp*, 5 *rpo*, 4 *pet*, 3 *chl* and 4 *ycf* genes. Furthermore, some genes appeared only once, such as the *rbc*, *cem*, *fts*, *clp*, *tuf*, and *ccs*. Significant differences were observed in genome size, GC content, total number of genes, number of *tRNA*s, number of introns, and number of protein-coding genes distributed on the positive and negative strands of the genome respectively. The chloroplast genome size ranged 150,157–223,902 bp. *Aphanochaete elegans* (HB201732) had the smallest chloroplast genome, and *Stigeoclonium helveticum* (UTEX 441) had the largest chloroplast genome. The GC content ranged 23.88–31.70%, of which *Aphanochaete elegans* (HB201732) had the lowest GC content, and *Chaetophoropsis polyrhium* (HB201646) had the highest GC content. The number of *tRNA*s ranged 25–30, which was markedly different. Introns varied between 2 and 33. *Aphanochaete elegans* (HB201732) only contained two introns, displaying the most compact genome, while *Schizomeris leibleinii* (UTEX LB 1228) contained 33 introns. Furthermore, the distribution of genes on the coding strand was skewed and varied among species. The protein-coding genes were distributed among both strands, and the number of genes at the plus or minus strand varied among different species. The distribution of protein-coding genes of *Aphanochaete elegans* (HB201732) was the most uneven (+/−, 51/16). The total length of the coding region accounted for 45.15–65.79%, and *Aphanochaete elegans* (HB201732) accounted for the highest proportion, while *Stigeoclonium* sp. (bmA10) accounted for the lowest proportion.

**Table 1:** The complete chloroplast genome features of the Chaetophorales

Taxa	Size (bp)	GC content (%)	Number of gene	CDS percent	CDS (plus/minus)		tRNA	rRNA	Intron
					+	–			
*Uronema confervicolum*	182,093	27.21	96	58.42	37	30	26	3	27
*Aphanochaete confervicola*	157,920	27.04	99	64.29	48	19	29	3	9
*Aphanochaete elegans*	150,157	23.88	99	65.79	51	16	29	3	2
*Chaetophora* sp.	208,126	30.27	98	49.87	47	20	28	3	14
*Stigeoclonium* sp.	193,940	27.92	98	45.15	23	44	28	3	28
*Chaetophoropsis polyrhizum*	214,786	31.70	95	45.68	27	40	25	3	26
*Draparnaldia mulabilis*	202,965	30.55	98	49.15	21	46	28	3	24
*Schizomeris leibleinii*	182,759	27.20	98	51.60	49	18	30	3	33
*Stigeoclonium helveticum*	223,902	28.90	97	48.60	24	43	28	3	21

Furthermore, five fragmentary chloroplast genomes were obtained. Despite different degrees of deletions in the chloroplast genome, partial genome sequences we generated, including complete sequences of all 58 protein-coding genes shared among the completely sequenced cpDNAs; therefore, protein-coding genes were maximally extracted for phylogenetic analyses (Table 1).

### Phylogenetic analyses based on the four nuclear concatenated markers (*18S* + *5.8S* + *ITS2* + partial *28S rDNA*)

**Fig. 1:**
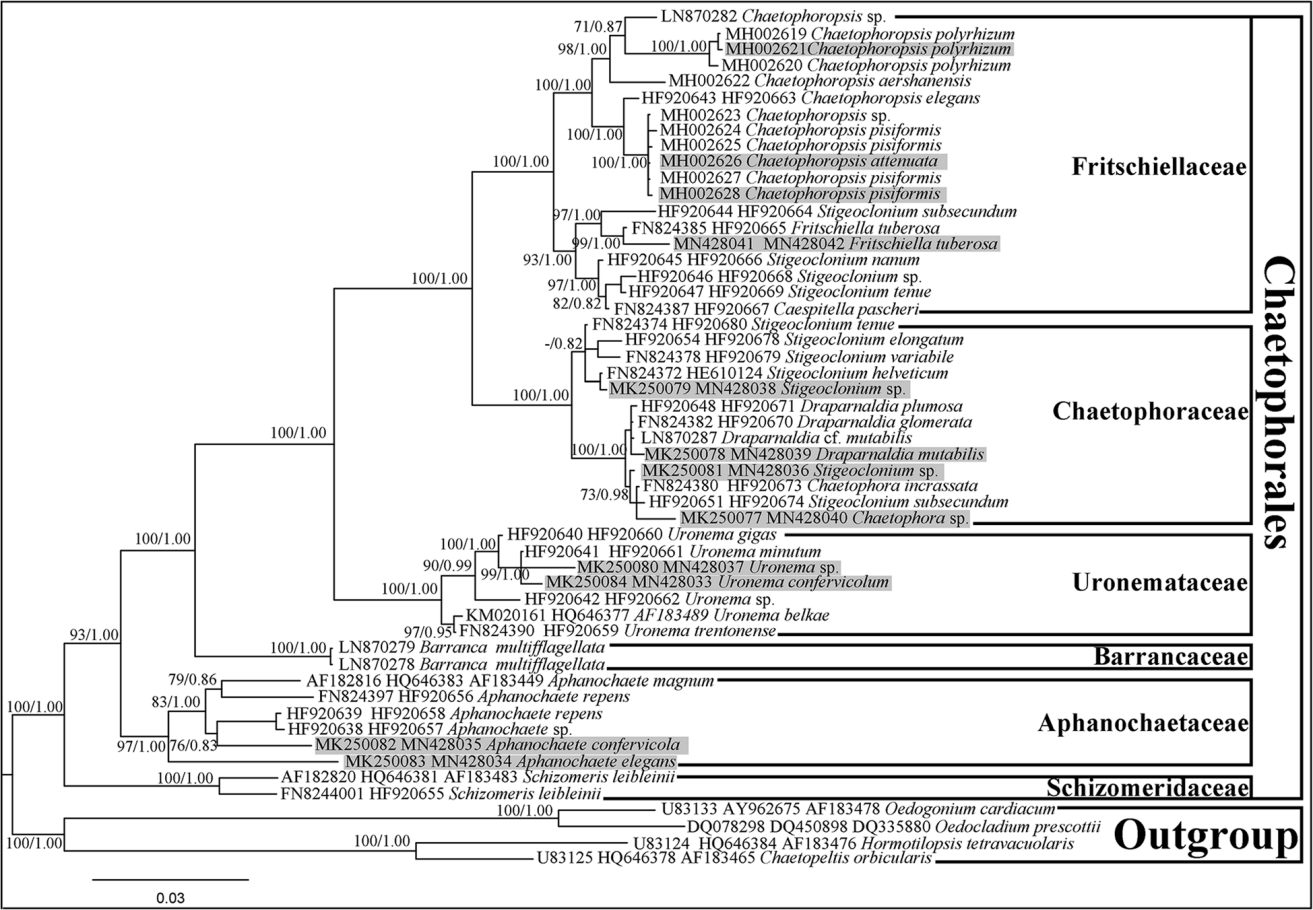
ML and Bayesian phylogenetic tree of the Chaetophorales constructed by using a concatenated data set of four markers (*18S* + *5.8S* + *ITS2* + partial *28S rDNA*). The best-fit model was GTR + I + G. The numbers on the nodes represent the posterior probabilities (PP)/bootstrap support values (BP) above 50/0.50. The tree was rooted with two species of Oedogoniales and Chaetopeltidales respectively. Strains for chloroplast genomes investigated in this study are shaded in grey

The 53-taxa alignment comprised 3032 bp. In total, 664 sites among these nucleotides were variable, of which 496 sites were parsimoniously informative and 168 sites were singleton sites. The average content of A, T, C, and G was 24.17, 25.67, 21.46, and 28.70%, respectively, of which the G + C content (50.16%) was greater than that of the A + T content (49.84%). The transition/transversion ratio was 1.77. Chloroplast genomes from 12 strains represented four families herein and are shaded in grey. The phylogenetic trees generated using the Bayesian and ML methods displayed similar topologies to those reported previously [21, 39, 40]. Phylogenetic analyses of both alignments resolved six currently recognized monophyletic families in Chaetophorales [23]. Family Schizomeridaceae, as a sister family of those in Chaetophorales, was the basal clade of Chaetophorales with robust support (100/1.00) and was markedly separated from Aphanochaetaceae (Fig. 1).

### Phylogenetic analyses based on the chloroplast protein-coding genes

Both data sets were assembled from the following 58 protein-coding genes: *atpA, atpB, atpE, atpF, atpH, atpI, ccsA, cemA, chlB, chlN, clpP, petB, petD, petG, petL, psaA, psaB, psaC, psaJ, psbA, psbB, psbC, psbD, psbE, psbF, psbH, psbI, psbJ, psbK, psbL, psbM, psbN, psbT, psbZ, rbcL, rpl2, rpl5, rpl14, rpl16, rpl20, rpl23, rpl36, rpoA, rpoC2, rps3, rps4, rps7, rps8, rps9, rps11, rps12, rps14, rps18, rps19, tufA, ycf12, ycf3,* and *ycf4.*

These aforementioned genes formed a concatenated nucleotide (nt) dataset comprising 32,019 and 21,346 base pairs (without 3rd codon positions). In total, 18,578 sites and 10,706 in these nucleotides were variable, of which 16,752 and 9429 sites were parsimoniously informative and 1826 and 1277 sites were singleton sites. The average content of A, T, C, and G was 31.44, 33.89, 15.25, and 19.42% for the complete data set, and 29.71, 32.57, 16.03, and 21.69% for the dataset without 3rd codon positions, wherein the A + T content was markedly greater than that of G + C. The 58 protein-coding genes concatenated amino acid (aa) dataset comprised 10,673 characters.

**Fig. 2:**
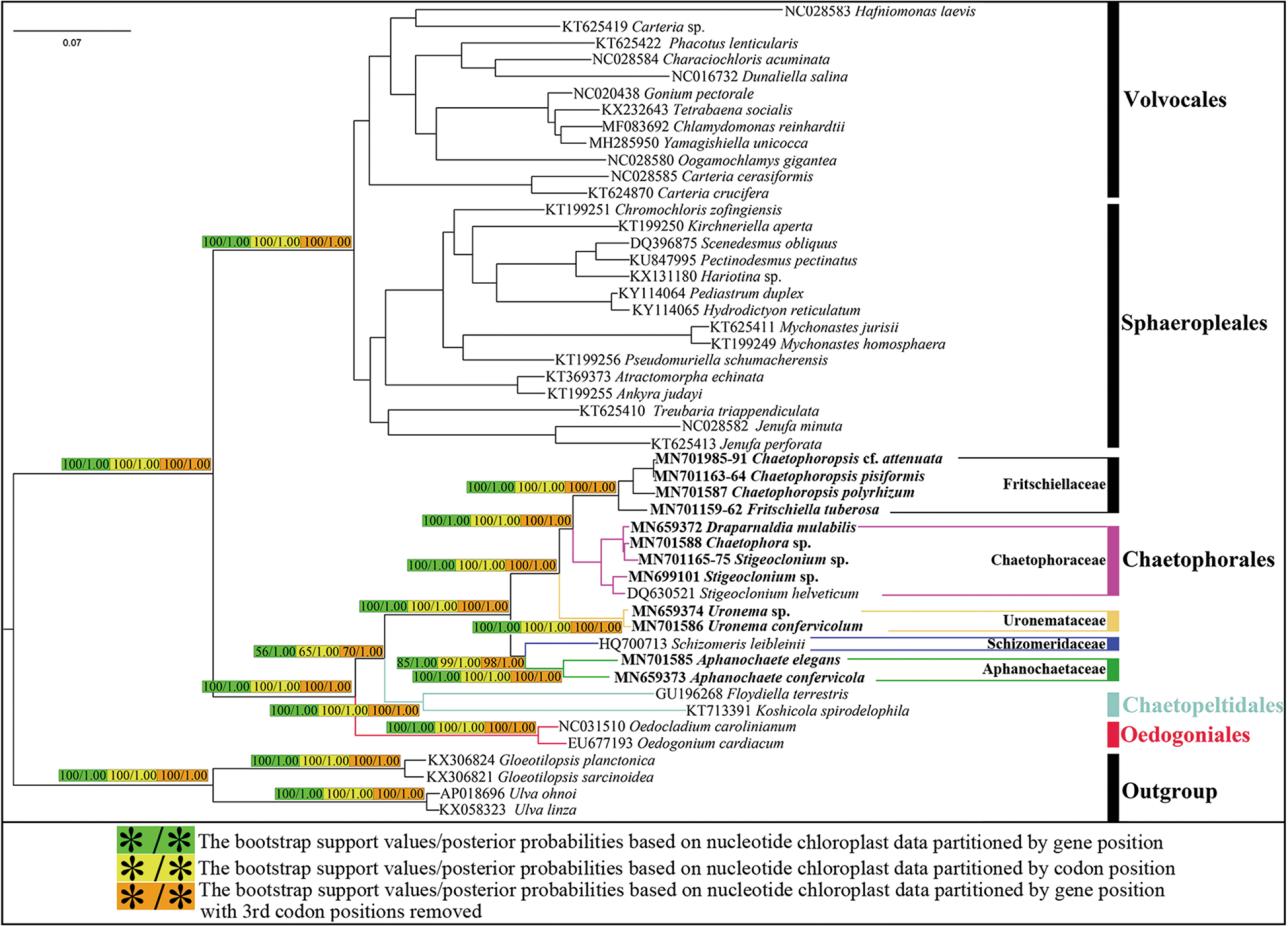
ML and Bayesian phylogenetic tree of the Chlorophyceae constructed by using 58 protein-coding genes of the chloroplast genomes. The concatenated nucleotide (nt) data set treated by three methods (partitioned by gene position, codon position and gene position without 3rd codon positions). The posterior probabilities (PP)/bootstrap support values (BP) above 50/0.50 are only shown on the key nodes. The tree was rooted with four species of the Ulvophyceae. Strains for chloroplast genomes investigated in this study are in bold

Maximum likelihood (ML) phylogenetic trees generated with the concatenated nucleotide (nt) data set treated with three methods (partitioned by gene position, codon position, and gene position without 3rd codon positions) had low support values at the node of the clade (orders Chaetophorales and Chaetopeltidales) (56/65/70) (Fig. 2).

**Fig. 3:**
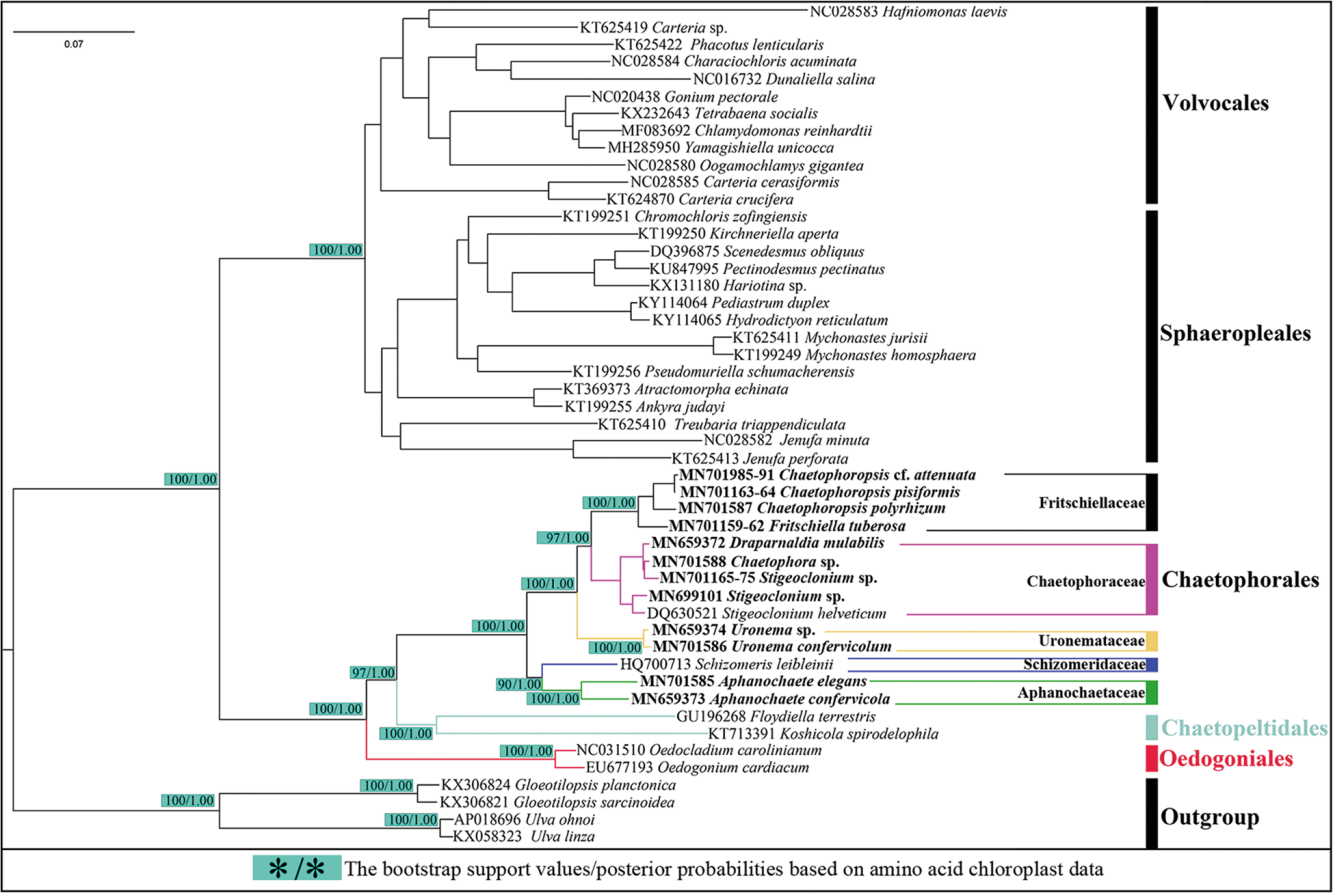
ML and Bayesian phylogenetic tree of the Chlorophyceae constructed by using concatenated 58 amino acid (aa) data set of the chloroplast genomes. The posterior probabilities (PP)/bootstrap support values (BP) above 50/0.50 are only shown on the key nodes. The tree was rooted with four species of the Ulvophyceae. Strains for chloroplast genomes investigated in this study are in bold

Nonetheless, the topologies of phylogenetic trees generated with concatenated datasets (nt and aa) were almost identical to each other and the support values in the amino acid (aa) data set were high at almost all nodes (Fig. 3), in contrast with previous studies with *rDNA* datasets [20, 21, 39]; this can be visualized on the basis of two aspects: the topologies and the support value, especially in the OCC clade*.* The support values in concatenated datasets of the chloroplast were markedly higher than those on *rDNA* datasets. Chlorophyceae diverged into two well*-*supported clades: VS and OCC clades. In the OCC clade, Oedogoniales was located at the base of the branch, and Chaetophorales and Chaetopeltidales were most closely related. Regarding the marked differences in the inner branching in Chaetophorales, Chaetophorales diverged into four well*-*supported clades, including five currently approved families except for Barrancaceae: Schizomeriaceae, Aphanochaetaceae, Uronemataceae, Fritschiellaceae, and Chaetophoraceae. Schizomeriaceae and Aphanochaetaceae could not be adequately separated, as *rDNA* datasets instead clustered into one branch at the base of order Chaetophorales. Chaetophoraceae sensu *lato* was located at the top branch of the Chaetophorales, displaying a basal split into the two well-supported clades, representing Fritschiellaceae and Chaetophoraceae sensu stricto, respectively. Family Uronemataceae as the sister was most closely related to Chaetophoraceae sensu *lato.*

### Synteny analysis

**Fig. 4:**
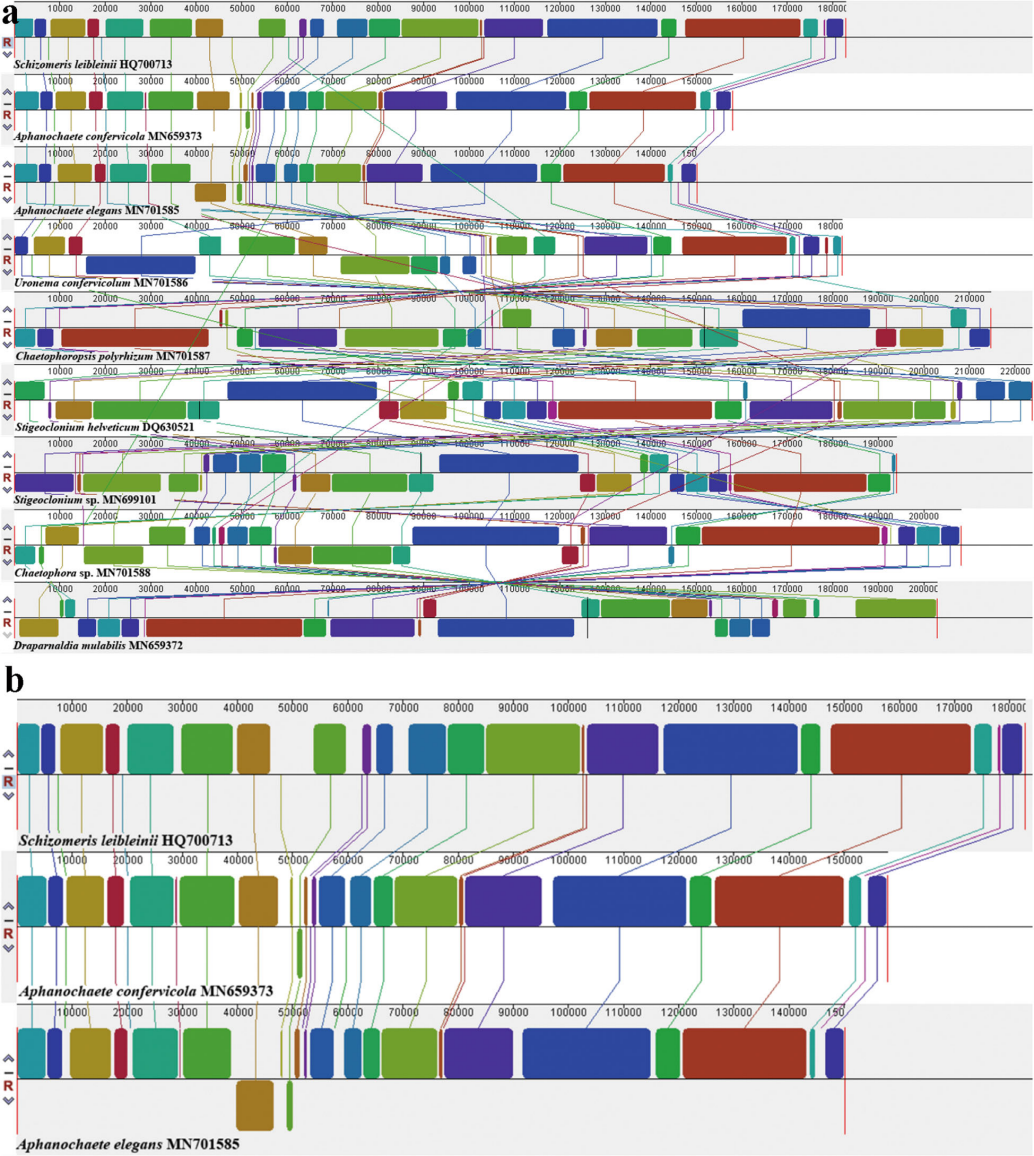
Synteny comparison of the Chaetophorales chloroplast genomes using progressiveMauve. **a**, Synteny comparison of nine chloroplast genomes representing five family; **b**, Synteny comparison of the family Schizomeridaceae (*Schizomeris leibleinii* HQ700713) and Aphanochaetaceae (*Aphanochaete confervicola* MN659373; *Aphanochaete elegans* MN701585). The coloured syntenic blocks are local collinear blocks; blocks above the centre line indicate they are on the same strand, and blocks below the centre line indicate they are on the opposite strand

**Fig. 5:**
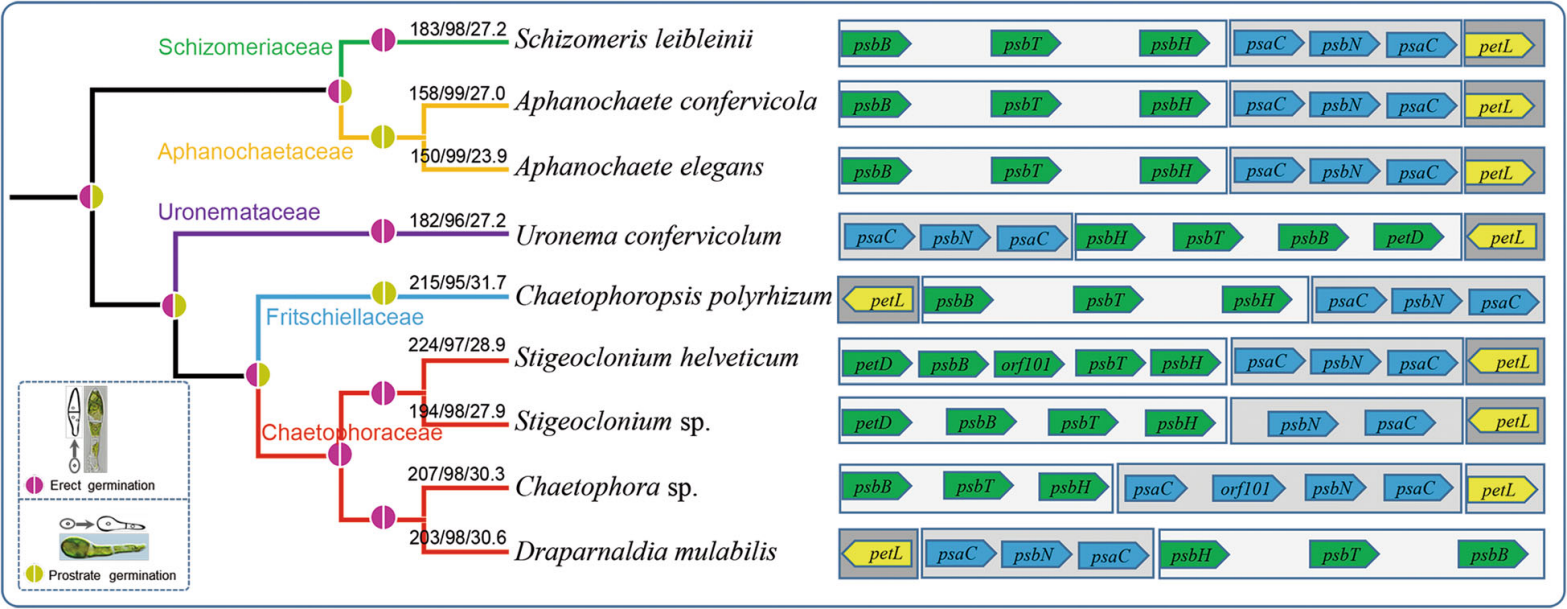
Chloroplast genomic feature and evolutionary relationship based on the zoospore germination of the Chaetophorales. The guide tree inferred from the chloroplast genomes by using progressiveMauve. Three conserved Locally Collinear Blocks (LCB) and genes of Chaetophorales are shaded in different colour. The numbers on the node represent the size/number of gene/GC content of the chloroplast respectively

ProgressiveMauve was used to analyze synteny in the chloroplast genome in Chaetophorales and set *Schizomeris leibleinii* as the reference genome [26]. Synteny analysis is illustrated in Fig. 4. Nine genomes from five families were used, including seven genera, and more than 27 locally collinear blocks (LCBs) were identified. The LCB connecting lines were confounding among chloroplast genomes and considerable rearrangements and inversions were noted, especially in Fritschiellaceae and Chaetophoraceae. The largest LCB was more than 40 kb (Fig. 4a). Synteny was highly homogenous among *Schizomeris leibleinii* (Schizomeridaceae), *Aphanochaete confervicola*, and *Aphanochaete elegans* (Aphanochaetaceae) (Fig. 4b). Three conserved LCBs comprising common genes (*psbB*, *psbT*, and *psbH*), (*psaC* and *psbN*), and (*petL*), respectively, were somewhat modified within most members of Chaetophorales. For example, compared to *Schizomeris leibleinii*, LCB (*psbB*, *psbT*, *psbH*) included another gene *petD* and *orf101*, and gene *petL* was inverted in *Stigeoclonium helveticum.* Similar patterns were observed in other species. Moreover, gene *psbN* was proximal to *psaC*; however, it did not split and transsplice *psaC* in *Stigeoclonium* sp. Nonetheless, the aforementioned three LCBs between Schizomeridaceae and Aphanochaetaceae were highly conserved (Fig. 5). Furthermore, the guide tree inferred from chloroplast genomes, using progressiveMauve, clearly indicated that Schizomeridaceae and Aphanochaetaceae clustered into one clade at the base of Chaetophorales (Fig. 5).

### Evolution of the Chaetophorales based on the germination type of zoospores

Morphological and life history observations clearly revealed that in the order Chaetophorales, zoospores of Schizomeriaceae contained zoospores for erect germination; Aphanochaetaceae, prostrate germination. Uronemataceae only contained zoospores for erect germination. In Chaetophoraceae sensu *lato*, zoospores of the family Chaetophoraceae sensu stricto and family Fritschiellaceae were present for erect germination and prostrate germination, respectively [23].

Based on the germination type of zoospores, the evolutionary hypothesis of Chaetophorales was proposed: the clade including Schizomeriaceae and Aphanochaetaceae including zoospores for erect and prostrate germination, respectively, was most closely related to the original ancestors of Chaetophorales, wherein the aforementioned two families were clustered together and located at the base of Chaetophorales; Uronemataceae displayed a loss of traits [20], only retaining zoospores for erect germination. In Chaetophoraceae sensu *lato*, the *Stigeoclonium*-like ancestors evolved independently in two directions. Some of them evolved into a group only with zoospores for prostrate germination and the highly differentiated prostrate in genera *Fritschiella* and *Chaetophoropsis* (Fritschiellaceae). The other part evolved into a group containing only zoospores for erect germination and the highly differentiated erect part in genus *Draparnaldia* (Chaetophoraceae) [41, 42], which were located at the top branch of Chaetophorales representing the most evolved taxa (Fig. 5).

## Disscussion

Unlike most green algae, the chloroplast genome of Chaetophorales does not have a typical quadripartite structure (a large single-copy region, a small single-copy region, and two inverted repeats separated by the single-copy region), and the inverted repeat region (IR) is obliterated. This phenomenon is not unique to species in Chaetophorales, some green algal groups have also presented a loss of this structure [6, 25, 43–49]. Inverted repeats have been lost numerous times during evolution in green algae, even in the same group [45]. Within Chlorophycean green algae, IR loss may be a synapomorphy marking the common ancestry of Chaetophorales and Chaetopeltidales [49], because the IR is obliterated in the plastomes of *Floydiella* (Chaetopeltidales), *Stigeoclonium*, and *Schizomeris* (Chaetophorales); however, it is present in Oedogoniales and other remaining investigated Chlorophyceae [5, 25, 26, 47]. The mechanisms leading to IR loss are still largely unknown [50].

In general, the size of the chloroplast genome of Chaetophorales tends to increase among families from Schizomeriaceae to Chaetophoraceae. The smallest chloroplast genome belongs to *Aphanochaete elegans* (family Aphanochaetaceae) and the largest one belongs to *Fritschiella tuberosa* (family Fritschiellaceae), despite its fragmentary chloroplast genomes. This difference in chloroplast genome size results primarily from differences in non-coding regions. Aerial or subaerial algae may have the larger chloroplast genomes than freshwater algae, e.g., *Fritschiella tuberosa* in this study, *Floydiella terrestris* in Chaetopeltidales [47] and *Trentepohlia odorata* in Trentepohliales [51]. Subaerial genera *Fritschiella and Floydiella* include only one species thus far [52]. Large genome constraints during speciation influence the species distribution and abundance, and plant physiology [53]. However, further studies are required to determine whether this phenomenon occurs in this order. Plant evolution in Chaetophorales has become more complex, consistent with that in the chloroplast genome, which tends to expand from the base to the top.

Evolution of the chloroplast genome in Chaetophorales tends to cause AT enrichment, consistent with other green algal groups [6]. In contrast with Chaetophorales plastomes, contiguous genes in the *Floydiella* chloroplast genome markedly tend to be clustered on the same strand [47]. The distribution of protein-coding genes in two chains of the chloroplast genome vary among different species; this distribution is most balanced in family Uronemataceae but gravely imbalanced in family Aphanochaetaceae, which can be explained by gene inversions and rearrangements [54]. Synteny analyses have accounted for numerous complex rearrangements and inversions among the chloroplast genomes of Chaetophorales; however, families Schizomeriaceae and Aphanochaetaceae displayed another trend. The plastome structures and conserved gene blocks in both Schizomeriaceae and Aphanochaetaceae were more similar to each other than to those of other families according to synteny comparison performed herein, as evident from their close phylogenetic relationship.

Phylogenetic analyses based on nuclear *rDNA* were incongruent with chloroplast genes, especially on the relative position of families Schizomeriaceae and Aphanochaetaceae, resulting from taxon sampling and characteristics of genes themselves. In contrast with numerous nuclear genes with limited resolving power and multi-copy in nature, thus potentially confounding phylogenetic reconstruction, organellar genes are typically single-copy by nature and do not present these issues [6, 55]. Furthermore, because of their relatively high and condensed gene content, chloroplast genomes are particularly useful for phylogenetic reconstruction [6]. Although support values of phylogenetic trees based on nt datasets were lower than those based on aa datasets at certain nodes, all of them supported the same unique topologies of the OCC clade and Chaetophorales.

Attempts to resolve relationships among the OCC clade met with limited success owing to the lack of samples and few genes [1, 2, 5, 20, 26, 47, 56–59]. By increasing the sizes of the chloroplast genomes of Chaetophorales, the present results confirmed that Chaetophorales and Chaetopeltidales constituted a clade, in contrast with basal Oedogoniales in the OCC group.

## Conclusions

In conclusion, chloroplast genome structure analyses, synteny analyses, and the zoospore germination analyses were concurrent with phylogenetic analyses based on the chloroplast genome, and all of them robustly determined the unique taxonomic scheme of Chaetophorales. Further studies are required to carry out phylogenetic analysis with a large number of samples to yield more convincing genomic data regarding phylogenetic relationships within Chaetophorales, thus furthering the current understanding of chloroplast genome evolution.

## Methods

### Taxon sampling and culture conditions

All strains described herein were sampled from different districts of China and voucher species was stored at the Freshwater Algal Herbarium (HBI), Institute of Hydrobiology, Chinese Academy of Sciences, Wuhan, China. Detailed information was given in Table 2.

**Table 2:** Detail information on the species with Chloroplast genome data of the order Chaetophorales. Taxa newly of this study are in bold

Family	Taxa	Isolator, isolation data	Voucher specimen	GenBank accession number		
				18S rDNA	5.8S-ITS2-partial 28S 28S	cpDNA
Schizomeridaceae	*Schizomeris leibleinii*	unknown	UTEX LB 1228	unkown	unkown	HQ700713 [26]
Aphanochaetaceae	***Aphanochaete confervicola***	B. W. Liu, 2017,. Hubei province, China. An epiphyte on the *Oedogonium*, freshwater.	HB201725	MK250082	MN428035	MN659373
	***Aphanochaete elegans***	B. W. Liu, 2017,. Hubei province, China, on the water grass, freshwater.	HB201732	MK250083	MN428034	MN701585
Uronemataceae	***Uronema*sp.**	B. W. Liu, 2017,. Guizhou province, China, on the Lotus leaf in a pool, freshwater.	GZ201706	MK250080	MN428037	MN659374
	***Uronema confervicolum***	B. W. Liu, 2017,. Henan province, China, on water grasses in a river, freshwater.	LY201701	MK250084	MN428033	MN701586
Chaetophoraceae	***Stigeoclonium*sp.**	B. W. Liu, 2016, Tibet, China, on the stones in a stream, freshwater.	bmA10	MK250079	MN428038	MN699101
	***Draparnaldia mulabilis***	B. W. Liu, 2017, Aershan, Hinggan, Inner mongolia province, China, on the stones in Halaha river, freshwater.	AES201713	MK250078	MN428039	MN659372
	***Chaetophora*sp.**	B. W. Liu, 2017, Aershan, Hinggan, Inner mongolia province, China, on the stones in Halaha river, freshwater.	AES201704	MK250077	MN428040	MN701588
	*Stigeoclonium helveticum*	unknown	UTEX 441	EU123941	unkown	DQ630521 [25]
	***Stigeoclonium*sp.**	B. W. Liu, 2016,. Hubei province, China. Wuhan Botanical Garden, Chinese Academy of Sciences, on the stick in a pool, freshwater.	HB201635	MK250081	MN428036	MN701165–75
Fritschiellaceae	***Fritschiella tuberosa***	B. W. Liu, 2018,. Hubei province, China. Sanbar of Yangtze River, on the moist soil.	HB201823	MN428041	MN428042	MN701159–62
	***Chaetophoropsis polyrhizum***	B. W. Liu, 2016,. Hubei province, China. Wuhan Botanical Garden, Chinese Academy of Sciences, on water grasses in a pool, freshwater.	HB201646	MF497328	MH002621	MN701587
	***Chaetophoropsis pisiformis***	B. W. Liu, 2017, Guizhou province, China. Shuangrufeng, on the Lotus leaf in a pool, freshwater.	XR201704	MH002618	MH002628	MN701163–64
	***Chaetophoropsis*cf.*attenuata***	B. W. Liu, 2016, Hubei province, China. Wuhan Botanical Garden, Chinese Academy of Sciences, on the stick in a pool, freshwater.	FHB201644	MH002616	MH002626	MN701985–91

Each sample was preserved in 4% formalin for the morphological study. Natural samples were isolated using an Olympus SZX7 microscope (Olympus Corp., Tokyo, Japan) and rinsed with double*-*distilled H_2_O. The algae were first grown in culture dishes on sterilized BBM medium [60] solidified with 1.2% agar under the photon fluence rate of 15–35 μmol m^− 2^ s^− 1^ in a 14:10 h light:dark cycle at 20 °C and transferred into the fresh medium every week untill to be the unialgal strains. The unialgal strains were cultivated in liquid BBM medium [60] at 20 °C, under the photon fluence rate of 45–60 μmol m^− 2^ s^− 1^, in a 14/10 h light/dark cycle. The algae were grown at 20 °C in the dark for approximately 48 h to induce the liberation of zoospores. Microphotographs were taken with an Olympus BX53 light microscope (Olympus Corp., Tokyo, Japan) using the differential interference contrast method. The photographs were taken under an oil immersion objective lens.

### Nuclear DNA extraction, polymerase chain reaction amplification and phylogenetic analyses

Nuclear DNA extraction and phylogenetic analyses were conducted according to Liu et al. [40]. The polymerase chain reaction (PCR) of the *18S rDNA* was amplified according to Medlin et al. [61]. Amplifications of internal transcribed spacer of nuclear ribosomal DNA (ITS) and of partial *28S rDNA* were performed using (1) EAF3 forw + ITS055 rev or (2) 1380 forw + 1495 rev [39]. ContigExpress Project (Invitrogen, Grand Island, New York USA) was used to edit low-quality regions and assemble the partial sequences.

### Chloroplast DNA sequencing, assembly, and annotation

Twelve species were used to isolate the chloroplast DNA. Chloroplast DNA (cpDNA) was isolated using an improved extraction method [62]. Purified DNA was fragmented and used to construct short-insert libraries according to the manufacturer’s instructions (Illumina), and sequenced on the Illumina Hiseq 4000 [63]. The data were trimmed using SOAPnuke 1.3.0 [64] and assembled with SPAdes 3.13.0 [65].

The chloroplast genes were annotated using an online DOGMA tool [66]. Protein-coding and ribosomal RNA genes were further polished using Blast with genes from the available the Chaetophorales Chloroplast DNA [25, 26]. The tRNA genes were redetected using tRNAScan-SE 1.21 [67]. The circular chloroplast genome maps were drawn using OrganellarGenomeDRAW 1.2 [68]. Intron boundaries were determined by modeling intron secondary structures and by comparing intron-containing genes with intronless homologs [69, 70]. The annotated chloroplast genomes were submitted to GenBank under the accession numbers given in Table 2.

### Phylogenetic analyses based on chloroplast genome

Total 49 Chlorophyceae (Chlorophyta) taxa were used to generate the analysed nucleotide and amino acid data sets. The large, hypervariable genes *ftsH*, *ycf1*, *rpoC1*, and *rps2* were not included in analyses, and only genes present in all ingroup and outgroup taxa were used for phylogenetic analyses [31].

Each gene was aligned using mafft 7.0 [71]. The ambiguously aligned regions were further manually edited and adjusted by eye and translated into amino acid using MEGA6 [72]. All genes were then concatenated using Phyutility [73]. The evolutionary models and partitioning of each data set were determined by PartitionFinder2 [74].

The concatenated data set of nucleotide were partitioned by gene position, codon position and gene position without 3rd codon positions respectively. The concatenated data set of amino acid was partitioned by gene position. Phylogenies were inferred from both data sets using maximum likelihood (ML) and Bayesian (BI) methods. Phylogenetic trees were conducted using MrBayes 3.2.6 [75] and RAxML 8.2.10 [76]. MrBayes was ran for 5,000,000 generations, sampling and printing every 500 and bootstrap analyses with 1000 replicates of the ML dataset were performed to estimate the statistical reliability. The synteny comparison was visualized using progressiveMauve [77].

## Supplementary information

**Additional file 1 Figs. S1-S7.** Gene map of seven complete chloroplast genomes of the Chaetophorales. Arrows show the direction of transcription. The same colour block shows the functional gene group (legend at bottom left). Transfer RNAs are represented by their one-letter amino acid code. The grey circle on the inside shows a graph of the GC content. **Fig. S1.***Aphanochaete repens* (HB201725). **Fig. S2.***Aphanochaete elegans* (HB201732). **Fig. S3.***Uronema repens* (LY201701)*.***Fig. S4.***Chaetophoropsis polyrhiza* (HB201646)*.***Fig. S5.***Stigeoclonium* sp. (bmA10)*.***Fig. S6.***Draparnaldia mutabilis* (AES201713)*.***Fig. S7.***Chaetophora* sp. (AES201704).

## Data Availability

All data generated and analysed during this study are included in this published article and its supplementary information files. Raw sequencing data of all species are available from the National Center for Biotechnology Information (NCBI) (https://www.ncbi.nlm.nih.gov/). Accession numbers of cpDNA: MN659373, MN701585, MN659374, MN701586, MN699101, MN659372, MN701588, MN701165-MN701175, MN701159-MN701162, MN701587, MN701163-MN701164 and MN701985-MN701991.

## Abbreviations

SV clade: Sphaeropleales and volvocales
OCC clade: Oedogoniales, chaetophorales, and chaetopeltidales
IR: Inverted repeats
nt: Nucleotide
aa: Amino acid
ML: Maximum likelihood
HBI: Freshwater algal herbarium
PCR: Polymerase chain reaction
ITS: Internal transcribed spacer of nuclear ribosomal DNA
cpDNA: Chloroplast DNA
