# Supplementary material for: Taxonomic scheme of the order Chaetophorales (Chlorophyceae, Chlorophyta) based on chloroplast genomes
Source: BMC Genomics. 2020 Jun 26;21:442. doi: 10.1186/s12864-020-06845-y (PMC7320567; doi:10.1186/s12864-020-06845-y)
Supplement: Supplementary file 1 — Additional file 1 Figs. S1-S7. Gene map of seven complete chloroplast genomes of the Chaetophorales. Arrows show the direction of transcription. The same colour block shows the functional gene group (legend at bottom left). Transfer RNAs are represented by their one-letter amino acid code. The grey circle on the inside shows a graph of the GC content. Fig. S1.Aphanochaete repens (HB201725). Fig. S2.Aphanochaete elegans (HB201732). Fig. S3.Uronema repens (LY201701).Fig. S4.Chaetophoropsis polyrhiza (HB201646).Fig. S5.Stigeoclonium sp. (bmA10).Fig. S6.Draparnaldia mutabilis (AES201713).Fig. S7.Chaetophora sp. (AES201704). [file 12864_2020_6845_MOESM1_ESM.pdf]

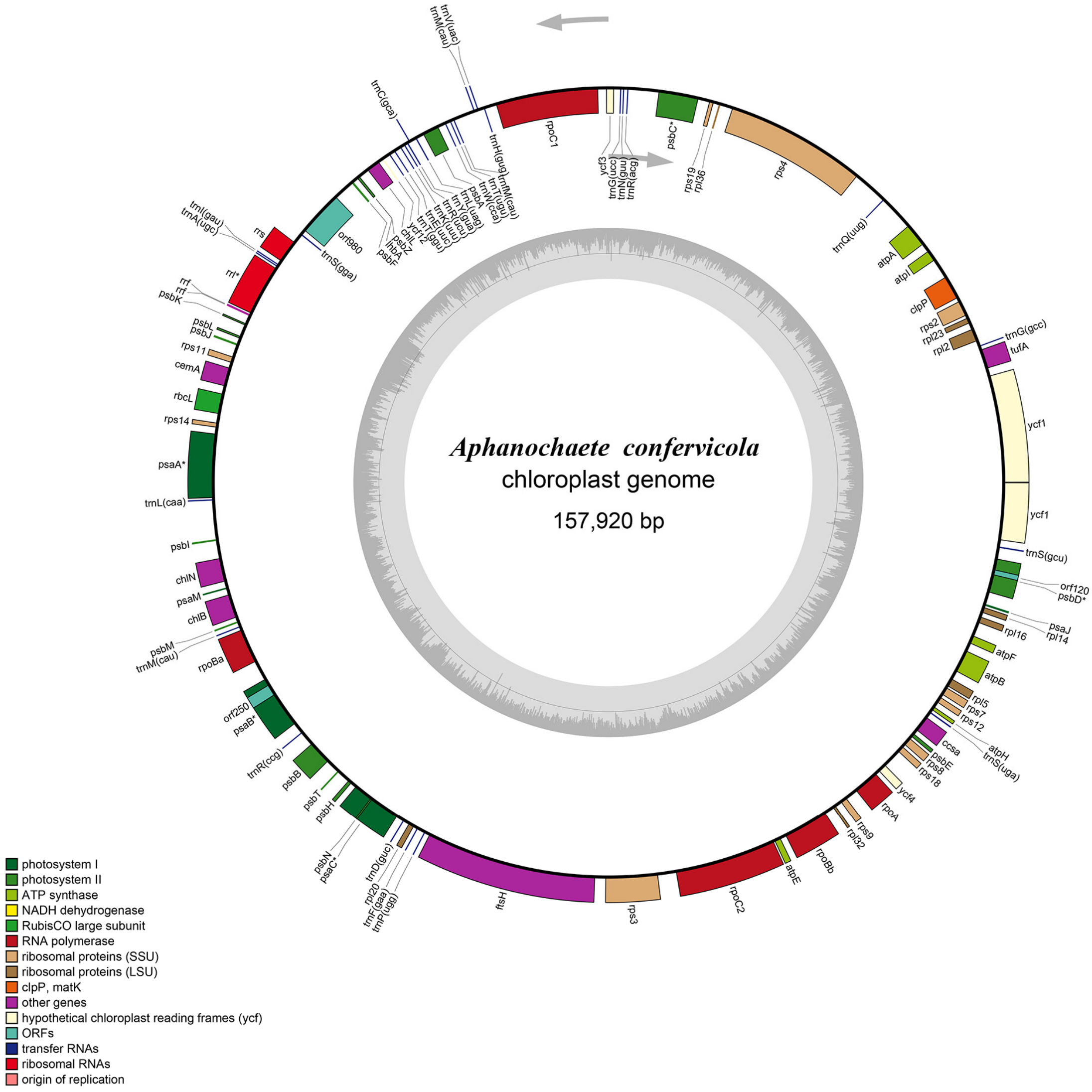

Figure S1 Chloroplast genome map of *Aphanochaete repens* (HB201725).

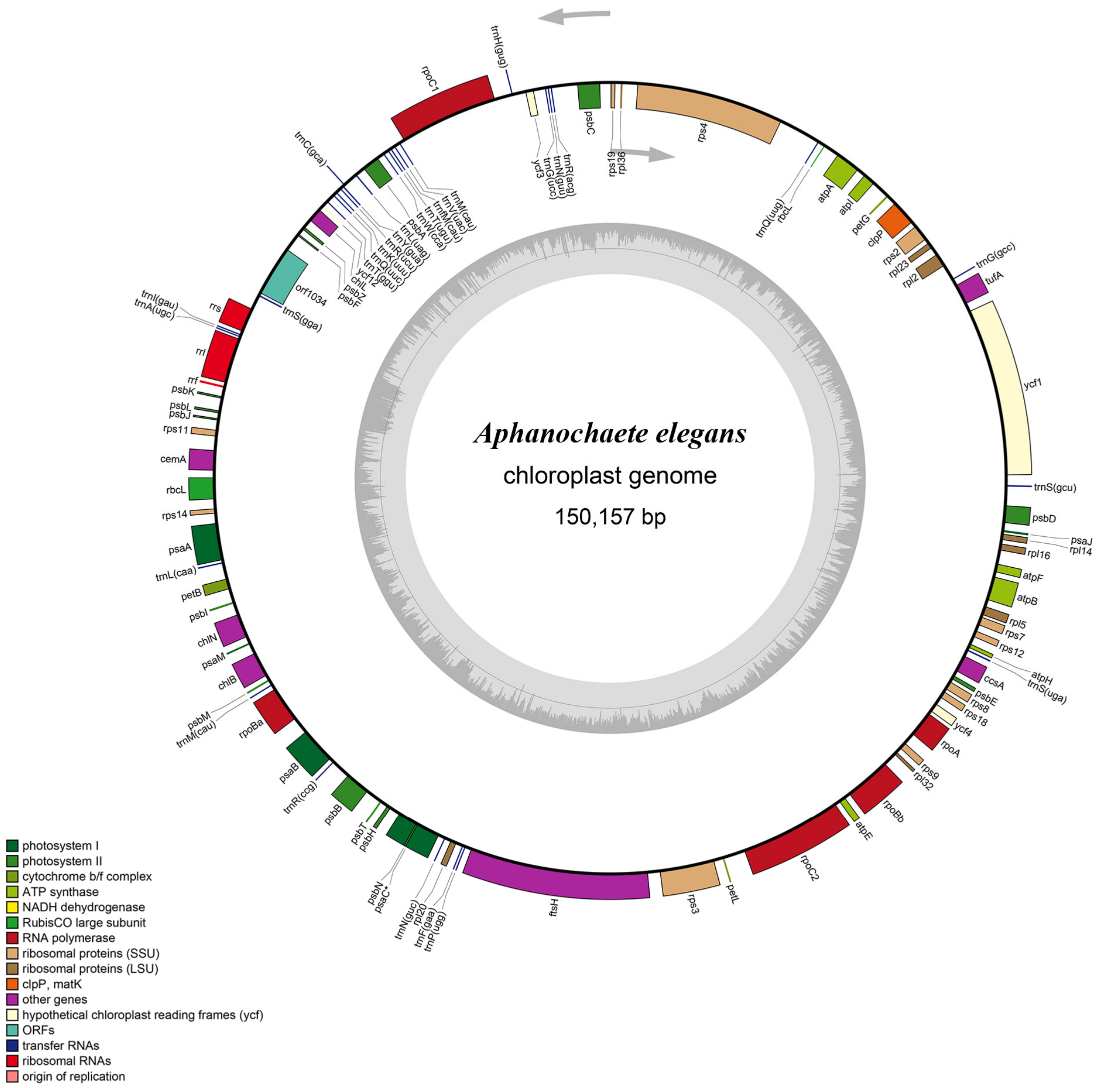

Figure S2 Chloroplast genome map of *Aphanochaete elegans* (HB201732).

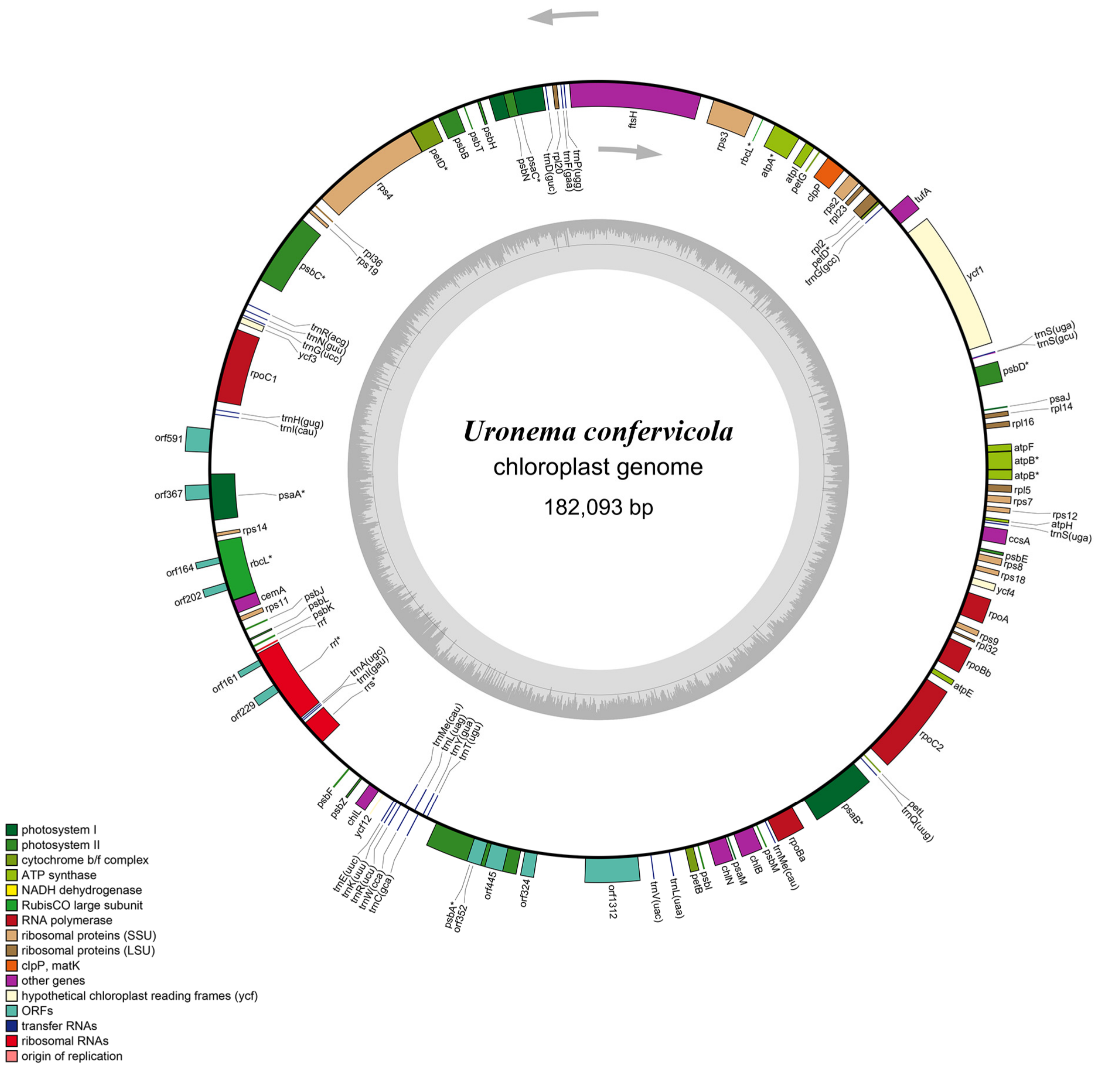

Figure S3 Chloroplast genome map of *Uronema repens* (LY201701).

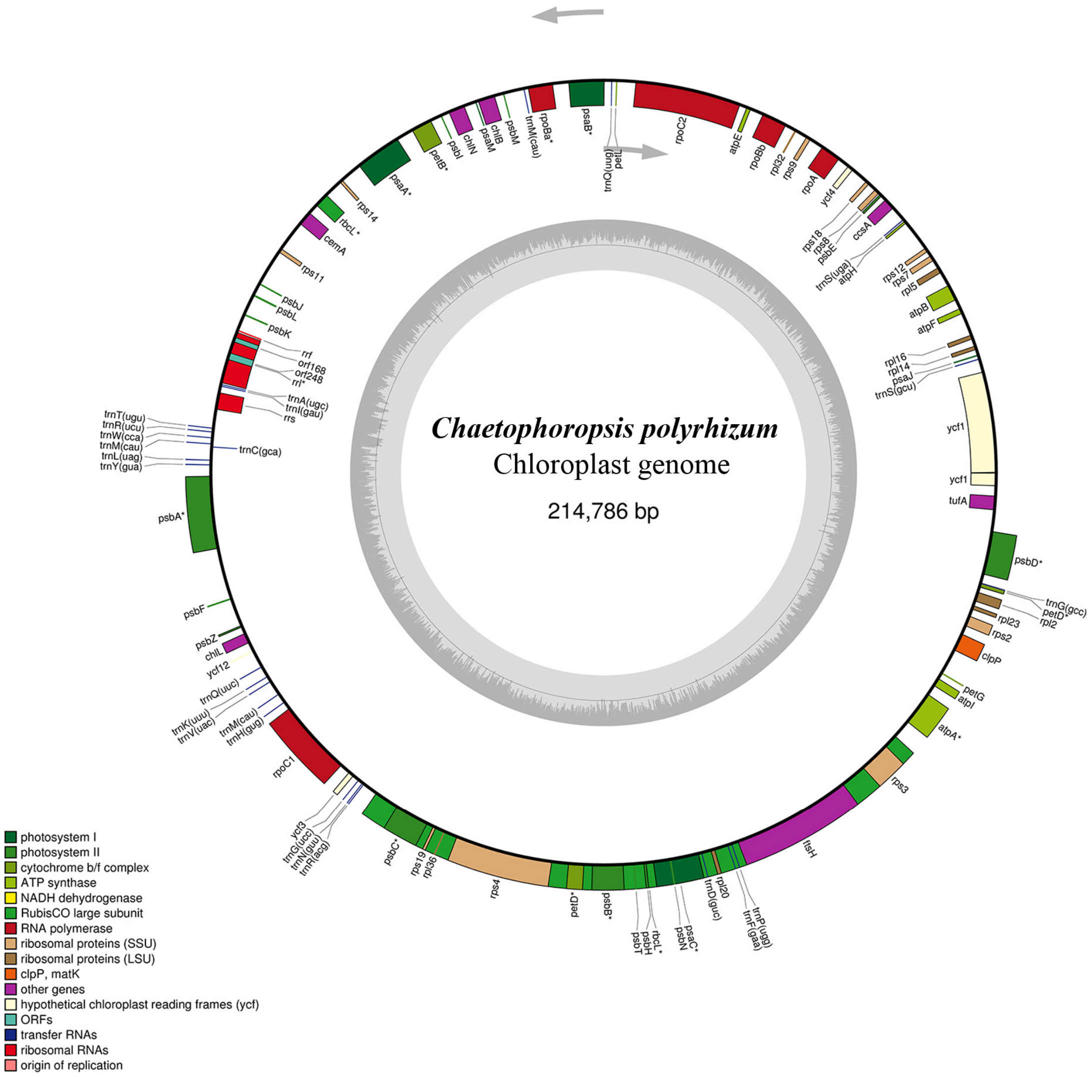

Figure S4 Chloroplast genome map of *Chaetophoropsis polyrhiza* (HB201646).

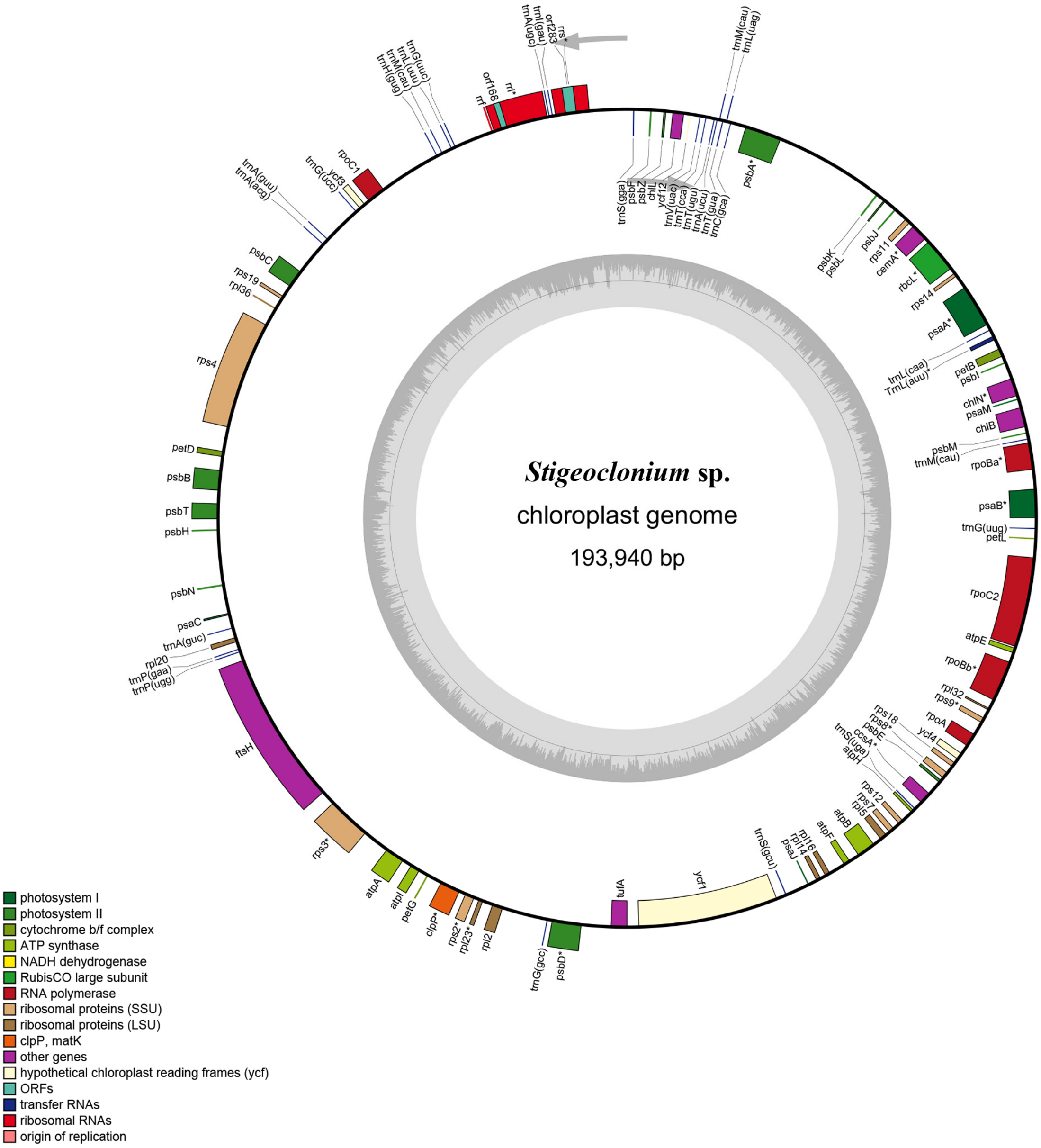

Figure S5 Chloroplast genome map of *Stigeoclonium* sp. (bmA10).

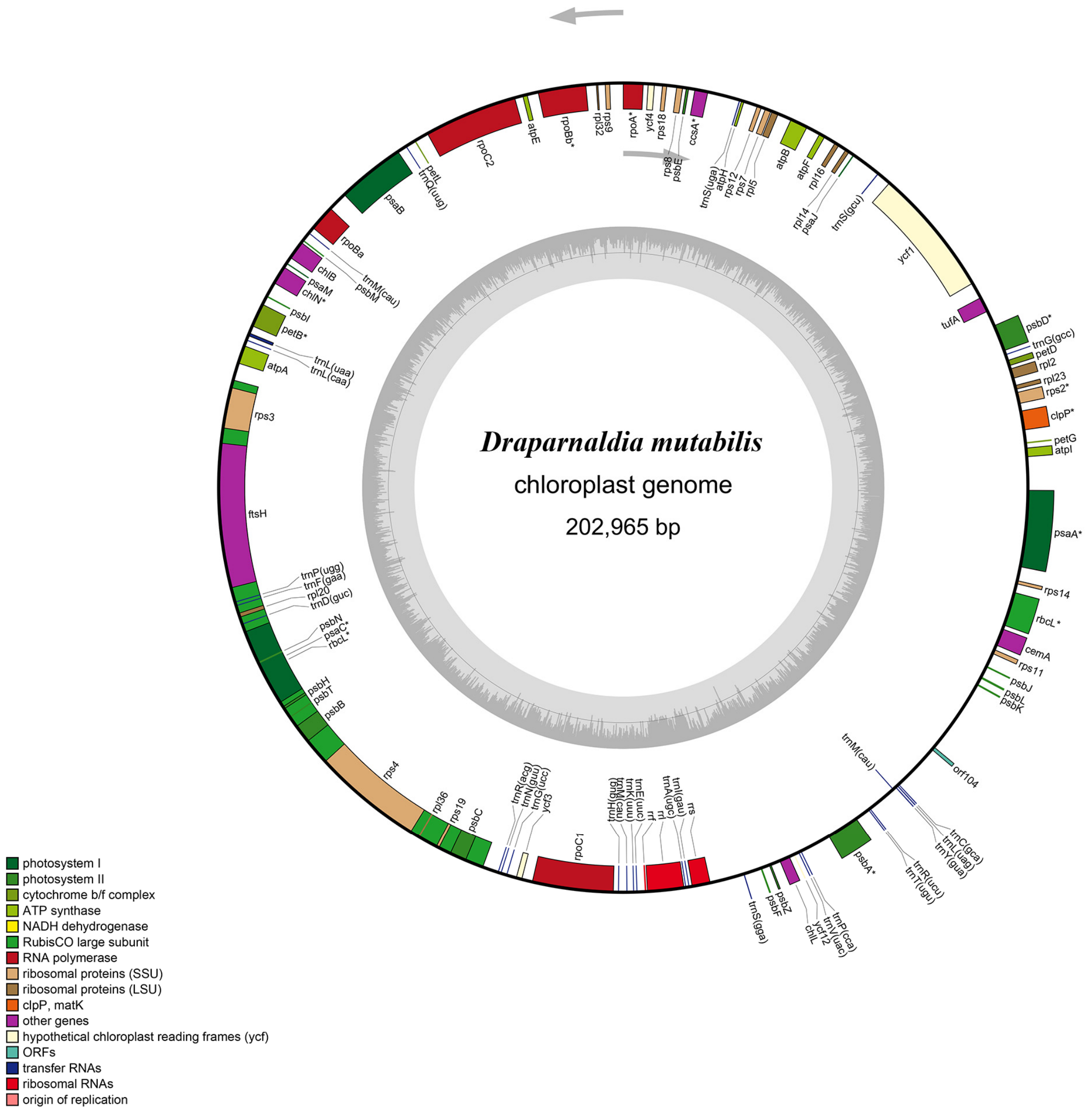

Figure S6 Chloroplast genome map of *Draparnaldia mutabilis* (AES201713).

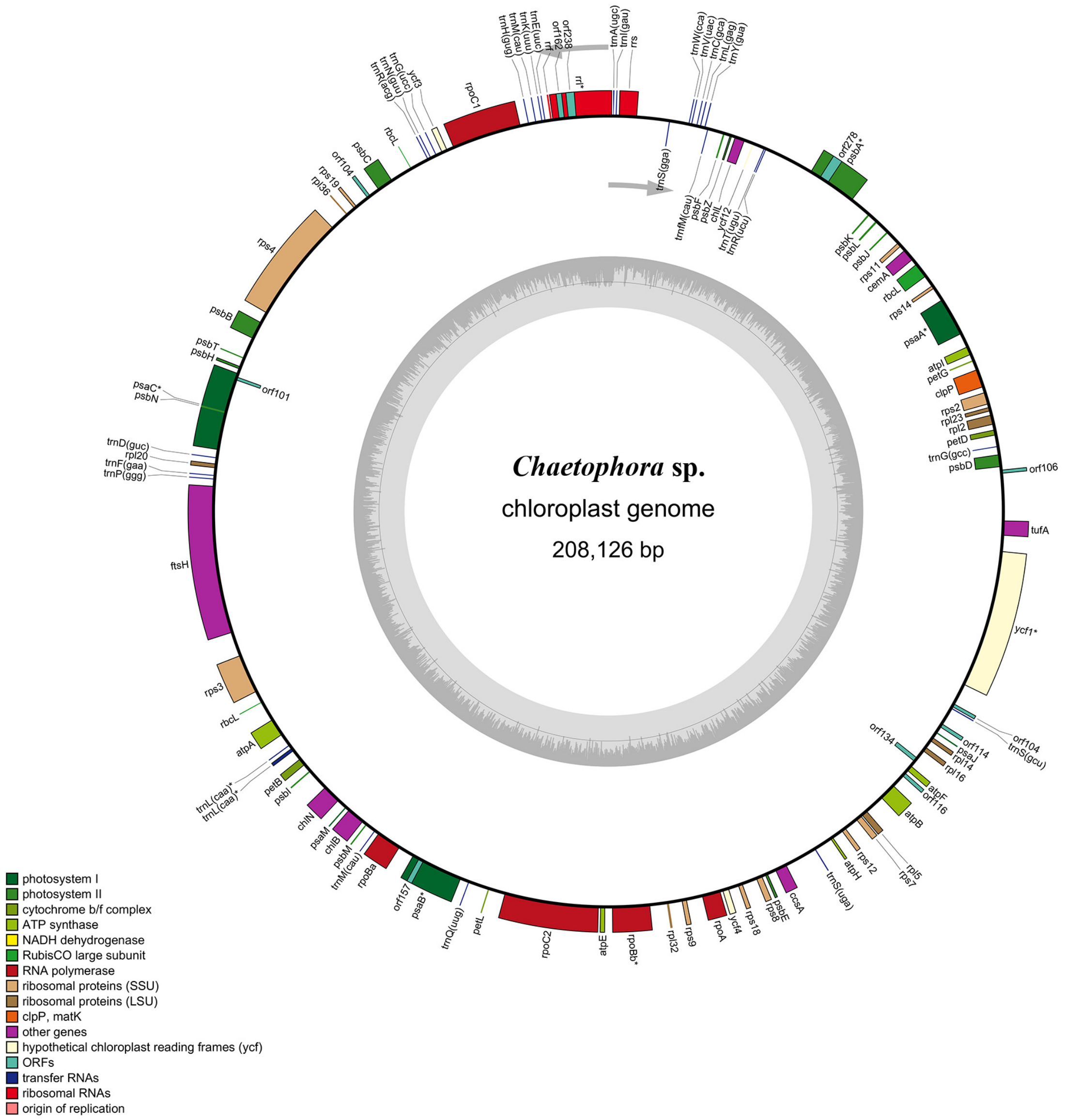

Figure S7 Chloroplast genome map of *Chaetophora* sp. (AES201704)
